# Rib fractures after chest compressions for cardiac arrest: retrospective analysis of the AfterROSC1 and AfterROSC2 multicenter databases

**DOI:** 10.1016/j.resplu.2025.100968

**Published:** 2025-04-30

**Authors:** Ivan Hemery-Allier, Wulfran Bougouin, Alain Cariou, Julien Lorber, Jeremy Bourenne, Francois Javaudin, Gwenhael Colin, Nicolas Chudeau, Marine Paul, Guillaume Geri, Jean Baptiste Lascarrou, AfterROSC Network

**Affiliations:** a Medical Intensive Care Unit, Saint-Nazaire Hospital, Saint-Nazaire, France; b AfterROSC Network Group, Paris, France; c Paris Cité University, INSERM, Paris Cardiovascular Research Center, Paris, France; d Mixed Intensive Care Unit, Jacques Cartier Hospital, Massy, France; e Medical Intensive Care Unit, Cochin University Hospital, AP-HP, Paris, France; f Emergency Intensive Care and Shock Control, La Timone University Hospital, AP-HM, Marseille, France; g Emergency Department, Nantes University Hospital, Nantes, France; h Medical Intensive Care Unit, Vendée Regional Hospital, La Roche-sur-Yon, France; i Medical Intensive Care Unit, Le Mans Hospital, Le Mans, France; j Medical Intensive Care Unit, Versailles Hospital, Le Chesnay, France; k Intensive Care Unit, Ambroise Paré Hospital, Neuilly-sur-Seine, France; l Nantes University Hospital, Movement-Interactions-Performance Laboratory (MIP), UR 4334, Medecine Intensive Reanimation, Nantes, France

**Keywords:** Cardiac arrest, Rib fracture, Chest-wall injury

## Abstract

**Purpose:**

External chest compressions for resuscitation after out-of-hospital cardiac arrest (OHCA) can cause rib fractures, which are best diagnosed by computed tomography (CT). We assessed the prevalence, management, and associations with outcomes of CT-documented rib fractures in patients with OHCA.

**Methods:**

We retrospectively analyzed data collected prospectively at five AfterROSC Network centers in 2020–2023. We included consecutive patients with return of spontaneous circulation and coma after non-traumatic OHCA who underwent CT within 6 h after admission. Rib fractures and other chest-wall injuries were recorded. Associations with the day-90 functional outcome were sought. Analgesic treatment was compared between patients with 0–2 vs. ≥3 rib fractures.

**Results:**

Of 2129 patients, 233 (11%) underwent chest CT, which showed at least one rib fracture in 116 (50%). The mean number of rib fractures was 2.4 ± 3.4 and the median was 0 [0–4]. One patient had clinical flail chest. In patients with ≥3 rib fractures, the mean modified Cardiac Arrest Hospital Prognosis (mCAHP) score was higher (91 ± 23 vs. 82 ± 25) and a favorable day-90 neurological outcome (modified Rankin Scale score 0–3) was significantly less common, even after adjustment on mCAHP (18% vs. 35%; adjusted odds ratio, 0.37 [0.19–0.72]; *P* = 0.003). Analgesic therapy was not significantly different between patients with 0–2 and ≥3 rib fractures.

**Conclusions:**

Rib fractures related to chest compressions are common in OHCA survivors. Having ≥3 rib fractures was associated with a poorer prognosis after adjustment on cardiac-arrest characteristics. The management of pain related to rib fractures may require reappraisal.

## Highlights

• Among ICU patients who had chest CT after cardiac arrest, 50% had rib fractures.
• In a third of cases, the number of rib fractures was three or more.
• Fewer patients with ≥3 rib fractures had good functional outcomes (mRankin scale).
• Analgesics were not different in patients with 0–2 vs. ≥3 rib fractures.

## Background

In Europe, over 250 000 cases of out-of-hospital cardiac arrest (OHCA) occur each year.^1^ Immediate cardiopulmonary resuscitation (CPR) including external chest compressions is crucial to ensure survival. Bystander CPR is started in approximately 60% of patients and is followed by the return of spontaneous circulation (ROSC) in about a third of cases.^2^ Many patients with ROSC after OHCA are comatose and require intensive-care-unit (ICU) admission. Among these ICU patients, 5% to 40% are alive with a favorable neurological outcome on day 90.^3^, ^4^

The International Liaison Committee On Resuscitation (ILCOR) recommends chest compressions at a frequency of 100–120/minute, at the lower third of the sternum, with about 5 to 6 cm of depression.^5^ However, this intervention may cause chest-wall injuries in 66% to 95% of patients.^6^ The most common injuries are isolated rib fractures (66%–83%) and sternal fractures (8%–30%). Hemothorax and pulmonary contusion are less common.^6^ Chest-wall injuries can impair gas exchange and lung compliance, resulting in hypoxemia and/or hypercapnia, which are associated with a poorer prognosis^7^. Moreover, pain from chest-wall injuries may interfere with the assessment of neurological prognosis and may require specific analgesic therapy.^8^, ^9^ Guidelines on the management of chest-wall injuries do not apply to CPR-related cases.^10^ In particular, patients admitted after OHCA often have severe consciousness impairment that makes noninvasive ventilation difficult and precludes pain-intensity assessment using numerical or verbal scales. Few large studies of CPR-related rib fractures confirmed by imaging are available. A meta-analysis with computed tomography (CT)-confirmed CPR-related injuries provided limited outcome data.^6^

The primary objective of this retrospective study of two prospectively established databases was to assess the prevalence of CT-confirmed rib fractures in comatose patients admitted to the ICU after OHCA or in-hospital cardiac arrest (IHCA), CPR, and ROSC. The secondary objectives were to determine whether outcomes differed between patients with 0–2 vs. ≥3 rib fractures and to assess analgesic therapy in patients with rib fractures.

## Methods

This study is reported according to the Strengthening The Reporting of OBservational studies in Epidemiology guidelines^11^ and to International Committee of Medical Journal Editors recommendations. Both databases used were registered on ClinicalTrial.gov before enrollment of the first patient (Cohorts AfterROSC-1, NCT04167891, AfterROSC-2, NCT05606809). This study was approved by the appropriate ethics committee (2019-A01378-49; CPP-SMIV 190901 and 2022-A01811-42; CPP-IDFI 251022). The study complied with the Declaration of Helsinki and with all relevant laws and institutional guidelines. Information about the study was delivered to each patient’s relatives. When no relative was immediately available, emergency inclusion was allowed according to French law, and consent requested from a relative as soon as available; the patient was informed as soon as competence was regained. Patients who then refused participation had their data removed from the database.

### Computed tomography settings

CT was performed according to a previously published algorithm^12^ then at the physician’s discretion. General post-ICU-admission care was delivered as previously published.^13^ Post-resuscitation shock was defined as a need for vasopressors for more than 6 h despite adequate fluid loading. Suspected early onset pneumonia (EOP) was defined as new infiltrates on the chest radiograph with at least two of the following criteria, within 48 h after invasive mechanical ventilation (iMV) initiation: body temperature greater than or equal to 38.5 °C or less than or equal to 35.5 °C, leukocytosis (>10,000/mm^3^) or leukopenia (<4,000/mm^3^, and purulent tracheobronchial aspirate; however, in patients receiving targeted temperature management, a single criterion was sufficient to define suspected EOP. In patients who met the predefined criteria and were still receiving iMV, bronchial sampling for semiquantitative cultures were performed. Ventilator-associated pneumonia (VAP) was defined by the same criteria as for EOP occurring more than 48 h after iMV initiation. VAP and EOP were treating according to each protocols in each participating ICU. Withdrawal of life-sustaining therapy was performed according to current guidelines^14^ and French neuroprognostication data.^15^

### Study design and population

The AfterROSC Network includes 34 ICUs in public and private university- and non-university hospitals in France and Belgium. Since August 1, 2020, AfterROSC has prospectively collected data from adults admitted to network ICUs after cardiac arrest.^3^, ^15^

For this study, we included patients admitted between registry inception and December 31, 2023, to any of five network ICUs (Paris, Nantes, Marseille, Le Mans, and La Roche-sur-Yon). Inclusion criteria were age 18 years or older, OHCA or IHCA followed by CPR and ROSC, and coma defined as a Glasgow Coma Scale score ≤ 8 at ICU admission or, if sedation was started earlier, immediately before sedation initiation. Exclusion criteria were unavailability of chest CT data obtained before or within 6 h after ICU admission, cardiac arrest related to trauma, and unavailability of data on analgesic therapy.

### Data collection

All data were collected by a dedicated study nurse or investigator at each participating center. The following variables were recorded: baseline clinical data and comorbidities; characteristics of cardiac arrest and resuscitation (including times from collapse to chest-compression initiation [no flow] and chest compression initiation to ROSC [low flow]); clinical and laboratory characteristics at ICU admission; treatments delivered in the ICU; ICU length of stay (LOS); iMV duration; and functional and vital status at ICU discharge and at hospital discharge. The last neurological evaluation was performed on day 90 using the modified Rankin scale (mRS).^16^

For each patient, a single investigator (IHA) visited each center and reviewed the chest CT findings and the analgesic treatments administered. This investigator reviewed transverse and sagittal images of each CT scan in the bone window to detect rib and/or sternal fractures. Before the study, an expert radiologist at the Nantes University Hospital provided this investigator with specific training in bone-window CT assessment for rib- and sternum-fracture detection. Strong inter-observer agreement has been demonstrated for this assessment.^17^

### Outcome measures

The primary outcome was the prevalence of rib fractures. The secondary outcomes included notably favorable day-90 neurological outcome, defined as an mRS score of 0 to 3 (no symptoms to moderate disability).^18^, ^19^

### Statistical analysis

We described categorical variables as count (percentage) and continuous variables as mean ± standard deviation if normally distributed and as median [interquartile range] otherwise. We divided the patients into two groups according to whether the number of rib fractures was 0–2 or ≥3, in accordance with guidelines.^10^ To compare groups, we applied Pearson’s chi-squared test or Fisher’s exact test, as appropriate, for categorical variables and the *t*-test or Mann-Wilcoxon rank-sum test, as appropriate, for continuous variables. Missing data were counted and the numbers reported in the tables.

We performed univariate analyses to look for associations linking rib-fracture group (0–2 vs. ≥3) to patient outcomes. We then repeated these analyses after adjustment on cardiac-arrest severity as assessed using the modified Cardiac Arrest Hospital Prognosis (mCAHP) score.^20^ Last, we repeated these analyses after adjustment on mCAHP and the Charlson Comorbidity Index. Secondary outcomes reported as cumulative incidences were compared using the Fine-and-Gray competing-risks approach,^21^ with death as the competing risk.

All tests were two-sided and *P* values < 0.05 were considered significant. The analyses were performed using STATA/SE 14.2 (Lakeway Drive, TX).

## Results

### Patients

Compared to the 1868 patients who did not undergo chest CT early after admission, the 233 included patients less often had a shockable rhythm, ST-elevation myocardial infarction, or a probable cardiac cause to the arrest; they more often developed post-resuscitation shock (Table 1). The proportion of patients with a good neurological outcome on day 90 did not differ significantly (30% vs. 31%).

**Table 1:** Characteristics of the included and non-included patients admitted to the ICU for coma after cardiac arrest and return of spontaneous circulation.

	Included (N = 233)	Not included (N = 1896)	
Male, n (%)	161 (69)	1346 (71)	0.55
Age, years, mean ± SD	59 ± 16	61 ± 15	0.01
BMI, median [IQR]	26 [23–30]	25 [22–29]	0.17
Charlson Comorbidity Index, median [IQR]	3 [1–4]	3 [1–5]	0.07
Location at OHCA, n (%)			
- Home	119 (51)	1000 (53)	
- Public place	81 (35)	599 (31)	0.70
- Hospital	33 (14)	297 (16)	
Witnessed cardiac arrest, n (%)	200 (86)	1610 (85)	0.79
Bystander CPR, n (%)	166 (71)	1279 (67)	0.57
Cardiac arrest after EMS arrival, n (%)	35 (15)	338 (18)	0.27
Shockable rhythm, n (%)	102 (44)	980 (51)	0.009
No-flow duration, min, median [IQR]	2 [0–8]	2 [0–5]	0.28
Low-flow duration, min, median [IQR]	20 [10–30]	20 [10–30]	0.17
Time to EMS call, min, median [IQR]	10 [3–15]	9 [3–15]	0.84
Epinephrine use, n (%)	162 (69)	1395 (73)	0.19
Epinephrine dose, mg, median [IQR]	1 [0–3]	2 [0–4]	0.17
First arterial pH, median [IQR]	7.25 [7.13–7.34]	7.26 [7.15–7.35]	0.19
mCAHP score, mean–SD	85 ± 25	87 ± 24	0.24
Post-resuscitation shock, n (%)	133 (57)	1026 (54)	<0.001
STEMI, n (%)	40 (17)	647 (34)	<0.001
Early invasive coronary intervention, n (%)	95 (40)	928 (49)	<0.001
Probable cardiac cause to the arrest, n (%)	111 (47)	1171 (62)	<0.001
Temperature management, n (%)			
- None	25 (10)	304 (16)	
- Avoiding fever	144 (62)	1124 (60)	0.17
- Targeted temperature at 32 °C–36 °C	64 (28)	457 (24)	
Alive at ICU discharge, n (%)	93 (40)	771 (40)	0.65
Alive on day 90, n (%)	81 (46)	672 (35)	0.97
Favorable day-90 functional outcome, n (%)^a^	69 (30)	591 (31)	0.35
Modified Rankin Scale, n (%)			
0	22 (9)	185 (10)	
1	32 (14)	247 (13)	
2	10 (4)	96 (5)	
3	5 (2)	63 (3)	−
4	7 (3)	37 (2)	
5	5 (2)	12 (<1)	
6	152 (65)	1256 (66)	

BMI: body mass index; CPR: cardiopulmonary resuscitation; EMS: emergency medical service; mCAHP: modified Cardiac Arrest Hospital Prognosis score; STEMI: ST-elevation myocardial infarction

a defined as a modified Rankin Scale score of 0 to 3.

**Table 2:** Comparison of patients with vs. without three or more rib fractures.

	≥3 rib fractures N = 81	0–2 rib fractures N = 152	*P* value
**Male, n (%)**	59 (73)	102 (67)	0.37
**Age, years, mean ± SD**	64 ± 13	56 ± 16	<0.001
**BMI, median [IQR]**	26 [23–29]	25 [22–29]	0.59
**Charlson Comorbidity Index, median [IQR]**	3 [2–5]	2 [0–4]	<0.001
**Time from cardiac arrest to chest CT, hours, median [IQR]**	3 [2–4]	3 [2–4]	0.051
**Number of rib fractures, median [IQR]**	5 [4–7]	0 [0–0]	
**Flail chest**^a^**, n (%)**	1 (1)	0	0.35
**Sternal fracture, n (%)**	33 (41)	21 (14)	<0.001
**Pneumothorax, n (%)**	5 (6)	0 (0)	0.02
**Hemothorax, n (%)**	5 (6)	1 (<1)	0.01
**AIS score, median [IQR]**	2 [1–2]	0 [0–1]	<0.001
**EOP or VAP, n (%)**	55 (68)	94 (62)	0.39
**No-flow duration, min, median [IQR]**	2 [0–8]	2 [0–5]	0.28
**Low-flow duration, min, median [IQR]**	20 [10–30]	20 [10–30]	0.17
**Epinephrine use, n (%)**	65 (80)	97 (64)	<0.001
**Epinephrine dose, mg, median [IQR]**	2 [1–3]	1 [0–3]	0.50
**First arterial pH, median [IQR]**	7.24 [7.12–7.32]	7.25 [7.14–7.36]	0.26
**mCAHP, mean ± SD**	91 ± 23	82 ± 25	0.02
**Post-resuscitation shock, n (%)**	51 (63)	82 (54)	0.16
**STEMI, n (%)**	12 (15)	28 (18)	0.47
**Early invasive coronary intervention, n (%)**^b^	28 (34)	67 (44)	0.22
**Probable cardiac cause of the arrest, n (%)**	35 (43)	76 (50)	0.43
**Temperature management, n (%)**			0.26
- ***None***	*10 (12)*	*15 (10)*	
- ***Avoiding fever***	*54 (67)*	*90 (59)*	
- ***Targeted temperature at 32 °C–36 °C***	*17 (21)*	*47 (31)*	
**Invasive mechanical ventilation duration, days, median IQR**	4 [2–6]	3 [2–7]	−
**Length of ICU stay, days, IQR**	5 [2–7]	5 [3–9]	−
**Alive at ICU discharge, n (%)**	21 (26)	72 (47)	0.001
**Alive on day 90, n (%)**	19 (23)	62 (40)	0.023
**Favorable day-90 functional outcome**^c^**, n (%)**	15 (18)	54 (35)	0.003
**Modified Rankin scale, n (%)**			−
**0**	7 (9)	15 (10)	
**1**	8 (10)	24 (16)	
**2**	0	10 (4)	
**3**	0	5 (2)	
**4**	4 (5)	3 (1)	
**5**	2 (2)	3 (1)	
**6**	60 (74)	92 (4)	

BMI: body mass index; AIS: Abbreviated Injury Scale; EOP: early-onset pneumonia; CPR: cardiopulmonary resuscitation; EMS: emergency medical service; mCAHP: modified Cardiac Arrest Hospital Prognosis scale score; STEMI: ST-elevation myocardial infarction

a Flail chest was diagnosed by clinical evaluation (paradoxical motion).

b defined as done within 6 h after the return of spontaneous circulation

c defined as a modified Rankin Scale score of 0 to 3.

**Fig. 1:**
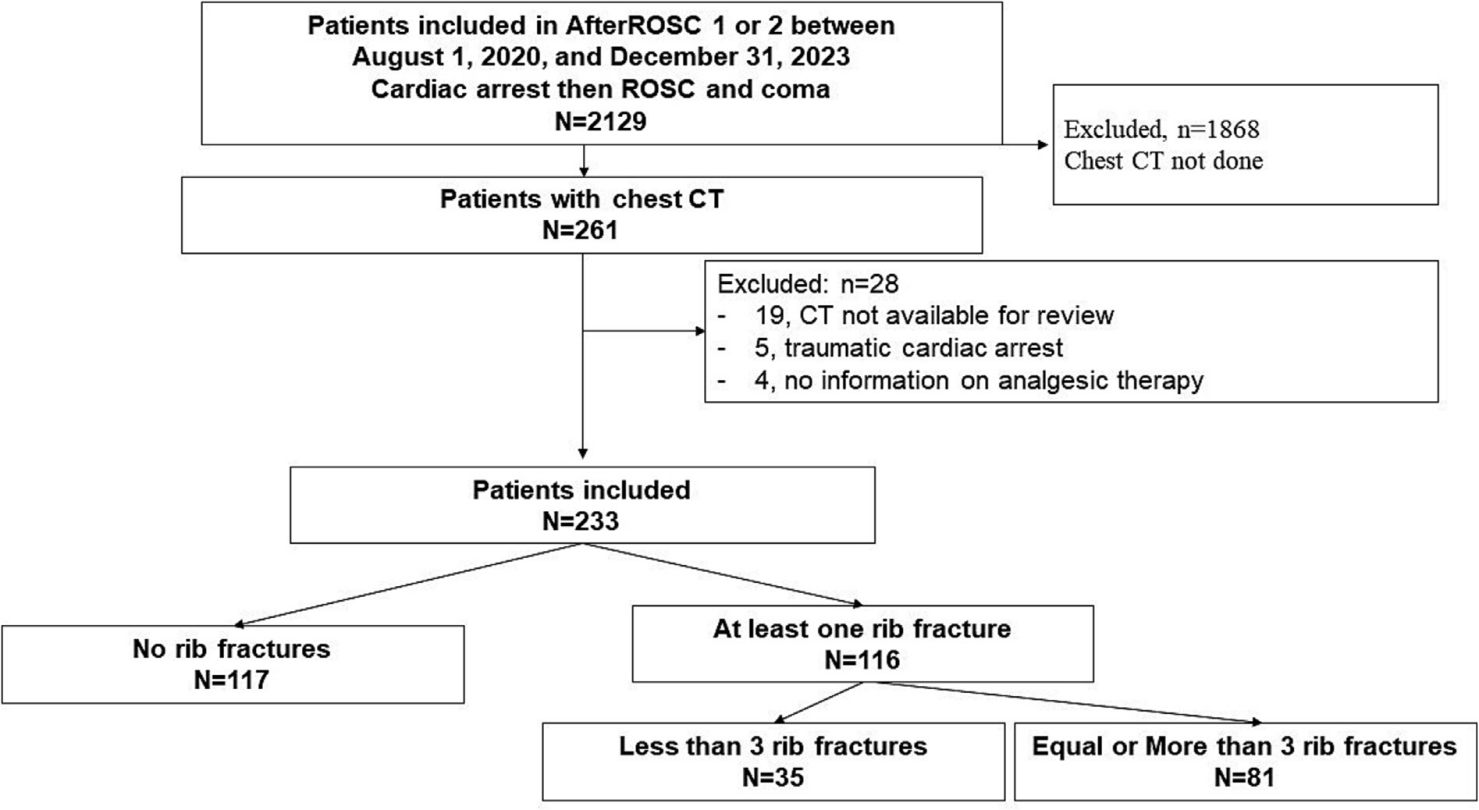
**Patient flowchart.** OHCA: out-of-hospital cardiac arrest; ROSC: return of spontaneous circulation; CT: computed tomography.

Fig. 1 is the patient flowchart. Of the 233 included patients, 117 had no rib fractures and 116 (49.8%) had at least one rib fracture. The patient features at baseline are listed in eAppendix 1 and eAppendix 2.

### Comparison of patients with 0–2 vs. ≥3 rib fractures (Table 2)

Patients with at least three rib fractures were older, had more comorbidities, and more often required epinephrine use during initial resuscitation. Consequently, their mCAHP score was significantly higher (91 ± 23 vs. 82 ± 25). They had more thoracic traumatic injuries, including more sternal fractures, pneumothorax, and hemothorax.

**Fig. 2:**
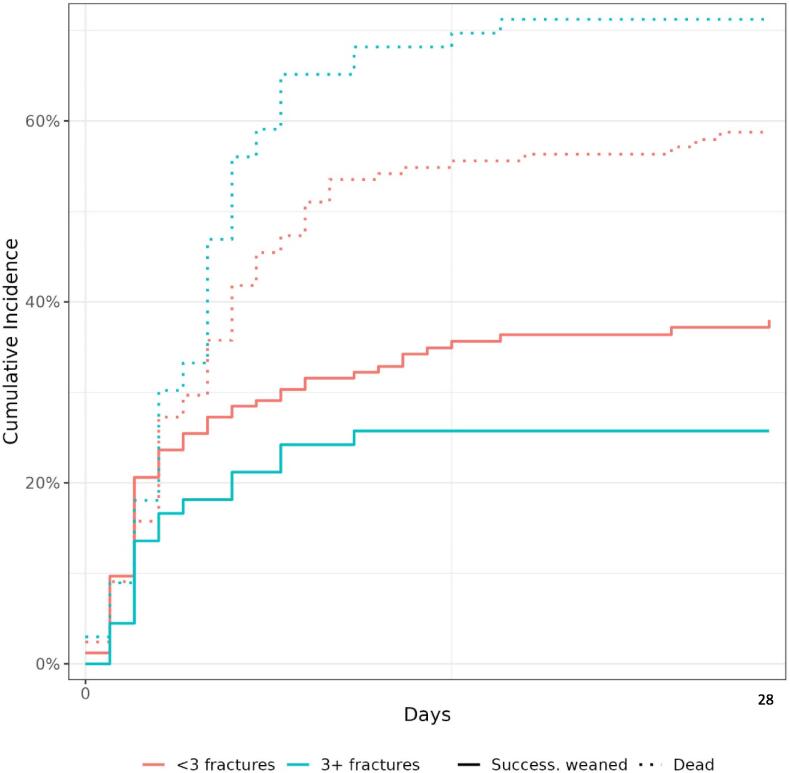
Kaplan Meier curve for survival and weaning off mechanical ventilation.

In this group, ICU and day-90 survival rates were lower and fewer patients had a favorable day-90 functional outcome (18% vs. 35%; *P* = 0.003). The association of three or more rib fractures with less often having a day-90 favorable outcome remained significant after adjustment on the mCAHP score (adjusted odds ratio, 0.37; 95% confidence interval, 0.19–0.72; *P* = 0.003) and after adjustment on the mCAHP and Charlson Comorbidity Index (adjusted odds ratio, 0.40; 95% confidence interval, 0.20–0.79; *P* = 0.008). The duration of iMV analyzed with death as a competing event was not significantly different between groups (*P* = 0.09, Fig. 2).

### Analgesic therapy in patients with 0–2 vs. ≥3 rib fractures

No differences in pain management were detected between patients with three or more rib fractures and other patients (Table 3). Locoregional anesthesia was performed in a single patient, and epidural anesthesia was performed in two patients in each group.

**Table 3:** Analgesia in patients with vs. without three or more rib fractures.

	≥3 rib fractures N = 81	0–2 rib fractures N = 152	*P* value
Acetaminophen, n (%)	46 (57)	82 (54)	0.19
NSAID therapy, n (%)	0 (0)	2 (1)	0.54
Tramadol, n (%)	4 (5)	4 (3)	0.45
Codeine, n (%)	0 (0)	1 (<1)	0.99
Nefopam, n (%)	9 (11)	14 (9)	0.65
Opioid by any route, n (%)	79 (98)	146 (96)	0.71
Opioid per os, n (%)	9 (11)	12 (8)	0.47
Opioid patch, n (%)	1 (1)	3 (2)	0.99
Opioid by PCA, n (%)	7 (8)	7 (5)	0.25
Opioid by continuous IV infusion, n (%)	79 (98)	143 (94)	0.34
- Number of days with continuous intravenous infusion	3 [2–4]	2 [2–4]	0.60
Epidural anesthesia, n (%)	2 (2)	2 (1)	0.17
Locoregional anesthesia, n (%)	0 (0)	1 (<1)	0.49
Ketamine infusion, n (%)	4 (5)	1 (<1)	0.051

IV: intravenous; NSAID: nonsteroidal anti-inflammatory drug; PCA: patient-controlled analgesia; VAP: ventilator-associated pneumonia.

## Discussion

Among patients who underwent chest CT after OHCA or IHCA, CPR, and ROSC and who were admitted to the ICU with persistent coma, half had at least one rib fracture. Patients with at least three rib fractures were older and had a heavier comorbidity burden compared to patients with 0–2 rib fractures. The group with three or more rib fractures had fewer patients alive at ICU discharge, alive on day 90, and having a favorable neurological outcome on day 90. These outcomes remained poorer after adjustment on the mCAHP score and comorbidities. Analgesic therapy was not significantly different between the group with at least three rib fractures and the group with 0–2 rib fractures, suggesting possible inadequate attention to analgesia.

Severe chest-wall injury defined as 3 or more rib fractures was significantly associated with day-90 functional prognosis. In a meta-analysis, many of the 74 included studies were done postmortem, precluding an evaluation of attributable mortality.^6^ A retrospective cohort study demonstrated an association of chest-wall injury severity with in-hospital mortality, in line with our findings.^22^ Importantly, in our cohort, this association persisted after adjustment on the mCAHP score, which includes CPR duration, a factor often deemed closely related to the risk of chest-wall injury. Mechanisms that may explain this association and constitute potential targets for interventions include impaired respiratory function, increased risk of lower-respiratory-tract infection, and worse systemic inflammation.^23^ However, in our cohort, neither EOP nor VAP were more common in the patients with three or more rib fractures.

Chest-wall injury is a major contributor to longer iMV duration, longer hospital LOS, and pain.^6^ However, in our cohort, iMV duration analyzed with death as a competing event was not longer in the group with three or more rib fractures. This finding may reflect insufficient sample size. Another possible explanation is our definition of severe chest-wall injury: in a previous study, iMV duration and hospital LOS were longer only in patients with at least six rib fractures.^22^

Pain-control methods and intensity did not differ significantly in our cohort between patients with three or more rib fractures and other patients. One possible explanation is that sedatives and opioids were given to nearly 90% of our patients as part of temperature management, irrespective of chest-wall injuries. However, separating sedative and opioid treatments given for neuroprotection as opposed to given for analgesia is challenging. Nonetheless, pain control may have been inadequate in patients who were doing well and were weaned off iMV. In this situation, aggressive management of rib fractures, including surgical procedures to optimize physical recovery and minimize pain, may be appropriate.^6^, ^24^ In patients with a poor neurological prognosis resulting in treatment-limitation decisions, optimal comfort including effective pain relief is essential. Of note, in chest-trauma patients who were not initially intubated, epidural analgesia did not decrease iMV needs, pain scores, or morphine needs.^25^ The increased frequency of chest-wall injuries seen after the recent change in guidelines indicates a need to improve injury detection and pain control.^26^

One limitation of our study is that we included only patients with chest CT. The criteria for obtaining this investigation were not standardized, resulting in a risk of selection bias. Two additional sources of bias are that all included patients were alive and were comatose upon arrival at the hospital.^27^ Second, the CT protocol was not uniform. Third, we did not use the Chest Wall Injury Society taxonomy for classifying the rib fractures.^28^ Instead, we followed French guidelines recommending that severe chest-wall injury be defined as three or more rib fractures.^10^ Fourth, the small sample size and the widespread use of sedatives and opioids as part of the neuroprotective strategy limited our ability to detect statistically significant differences in analgesic use between the two groups.

## conclusion

Chest-wall injuries, notably rib fractures, affected nearly half of patients who were admitted to the ICU for coma after cardiac arrest and who underwent early chest computed tomography. However, patients with early CT constituted only 11% of all post-cardiac-arrest patients admitted comatose to the ICU. Having three or more rib fractures was associated with worse outcomes. Finally, we provide indirect evidence that pain control may require improvement in patients with rib fractures after CPR.

## Author contributions

IHA, JL and JBL conceived and designed the study; analyzed and interpreted the data; drafted the manuscript; and revised the manuscript for important intellectual content.

All authors contributed to collect the study data, revised the manuscript for important intellectual content, and read and approved the final version of the manuscript.

## Consent for publication

Not applicable.

## CRediT authorship contribution statement

**Ivan Hemery-Allier:** Writing – review & editing, Writing – original draft, Methodology, Data curation, Conceptualization. **Wulfran Bougouin:** Writing – review & editing, Methodology, Investigation, Formal analysis. **Alain Cariou:** Writing – review & editing, Supervision, Methodology. **Julien Lorber:** Writing – review & editing, Formal analysis, Data curation, Conceptualization. **Jeremy Bourenne:** Writing – review & editing, Resources, Investigation. **Francois Javaudin:** Writing – review & editing, Visualization, Resources, Formal analysis. **Gwenhael Colin:** Writing – review & editing, Methodology, Investigation. **Nicolas Chudeau:** Writing – review & editing, Methodology, Investigation. **Marine Paul:** Writing – review & editing, Investigation, Conceptualization. **Guillaume Geri:** Writing – review & editing, Software, Methodology, Conceptualization. **Jean Baptiste Lascarrou:** Writing – review & editing, Software, Resources, Project administration, Funding acquisition, Formal analysis, Data curation, Conceptualization.

## Ethics approval

This study was approved by the appropriate ethics committee (2019-A01378-49; CPP-SMIV 190901 and 2022-A01811-42; CPP-IDFI 251022).

## Funding

This study was funded by a grant from the French Intensive Care Society (*Société de Réanimation de Langue Française*, SRLF) and by Ramsay Santé.

None of the funding sources had any role in designing the study; collecting, analyzing, or interpreting the data; writing the manuscript; or deciding to submit the manuscript for publication.

## Declaration of competing interest

The authors declare the following financial interests/personal relationships which may be considered as potential competing interests: JBL has received speaker fees from BD and Masimo. None of the other authors has any conflicts of interest to declare.
