# Supplementary material for: Rib fractures after chest compressions for cardiac arrest: retrospective analysis of the AfterROSC1 and AfterROSC2 multicenter databases
Source: Resusc Plus. 2025 Apr 30;24:100968. doi: 10.1016/j.resplu.2025.100968 (PMC12142319; doi:10.1016/j.resplu.2025.100968)
Supplement: Supplementary Data 1 [file mmc1.docx]

**eAppendix 1: Characteristics of the 233 patients who underwent chest computed tomography (CT) within 6 hours after ICU admission for coma after cardiac arrest and return of spontaneous circulation (Part 1)**

|  | **Patients with missing data** | **Overall cohort**  **N=233** |
| --- | --- | --- |
| Male, n (%) | 0 | 161 (69) |
| Age, years, mean±SD | 0 | 59±16 |
| BMI, median [IQR] | 1 | 25 [22–29] |
| Charlson Comorbidity Index, median [IQR] | 0 | 3 [1–4] |
| Location at cardiac arrest, n (%)  - Home  - Public place  - Hospital | 0 | 119  81  33 |
| Witnessed cardiac arrest, n (%) | 0 | 200 (86) |
| Bystander CPR, n (%) | 3 | 166 (71) |
| Cardiac arrest after EMS arrival, n (%) | 1 | 35 (15) |
| Shockable rhythm, n (%) | 1 | 102 (44) |
| No-flow duration, min, median [IQR] | 5 | 2 [0–86] |
| Low-flow duration, min, median [IQR] | 2 | 20 [10–30] |
| Time from telephone call to EMS arrival, min, median [IQR] | 25 | 10 [3–15] |
| Epinephrine use, n (%) | 0 | 162 (69) |
| Epinephrine dose, mg, median [IQR] | 0 | 1 [0–3] |
| First arterial pH, mean±SD | 6 | 7.25 [7.13–7.34] |
| Post-resuscitation shock, n (%) | 2 | 133 (57) |
| STEMI, n (%) | 1 | 40 (17) |
| Early invasive coronary intervention, n (%) | 26 | 95 (41) |
| Probable cardiac cause to the arrest, n (%) | 4 | 111 (48) |
| Temperature management, n (%)  - None  - Avoiding fever  - Targeted temperature at 32°C–36°C | 0 | 25  144  64 |
| Survival at ICU discharge, n (%) | 0 | 107 (46) |
| Survival on day 90, n (%) | 2 | 81 (35) |
| Favorable day-90 functional outcome^a^, n (%) | 9 | 69 (30) |

BMI: body mass index; CPR: cardiopulmonary resuscitation; EMS: emergency medical service; ICU: intensive care unit; STEMI: ST-elevation myocardial infarction

^a^defined as a modified Rankin Scale score of 0 to 3
