# Supplementary material for: Rib fractures after chest compressions for cardiac arrest: retrospective analysis of the AfterROSC1 and AfterROSC2 multicenter databases
Source: Resusc Plus. 2025 Apr 30;24:100968. doi: 10.1016/j.resplu.2025.100968 (PMC12142319; doi:10.1016/j.resplu.2025.100968)
Supplement: Supplementary Data 2 [file mmc2.docx]

|  | Patients with missing data | Overall cohort  N=233 |
| --- | --- | --- |
| Time from cardiac arrest to CT, min, median [IQR] | 71 | 3 [2–4] |
| Number of rib fractures per patient, median [IQR] | 0 | 0 [0–4] |
| At least one rib fracture, n | 0 | 116 |
| Flail chest, n | 0 | 1 |
| Sternal fracture, n | 0 | 54 |
| Pneumothorax, n | 0 | 5 |
| Hemothorax, n | 0 | 6 |
| Abbreviated Injury Scale score, median [IQR] | 0 | 1 [0–2] |
| EOP or VAP, n | 0 | 149 |
| Analgesics, n | 0 | 128 |
| *Acetaminophen* | *0* | *128* |
| *Non-steroidal anti-inflammatory drug* | *0* | *2* |
| *Tramadol* | *0* | *8* |
| *Codeine* | *0* | *1* |
| *Nefopam* | *0* | *23* |
| *Opioid* | *0* | *225* |
| Method of analgesic administration, n | 0 | 21 |
| *Oral* | *0* | *21* |
| *Opioid patch* | *0* | *4* |
| *Patient-controlled analgesia* | *1* | *14* |
| *Continuous intravenous opioid infusion*  *Duration, days, median [IQR]* | *0*  *0* | *222*  *3 [2–4]* |
| Epidural anesthesia, n | 0 | 4 |
| Locoregional anesthesia, n | 0 | 1 |
| Ketamine, n | 0 | 5 |
| Surgery, n | 0 | 0 |

**eAppendix 2: Characteristics of the 233 patients who underwent chest computed tomography (CT) within 6 hours after ICU admission for coma after cardiac arrest and return of spontaneous circulation (Part 2)**

EOP: early-onset pneumonia; VAP: ventilator-associated pneumonia
